# Assessing antibacterial, antiviral, and antifungal efficacy of non-porous materials using small droplet transfer: the simulated splash method

**DOI:** 10.1128/aem.02304-25

**Published:** 2026-04-01

**Authors:** Alexander J. Cunliffe, Emma Chareyre, Peter Askew, Gillian Iredale, Abby Marchant, James Redfern

**Affiliations:** 1 Department of Natural Sciences, Manchester Metropolitan University 5289, Manchester, United Kingdom; 2 Industrial Microbiological Services Ltd., Hartley Whitney, Hants, United Kingdom; Centers for Disease Control and Prevention, Atlanta, Georgia, USA

**Keywords:** antimicrobial materials, copper, relative humidity, small droplet, built environment

## Abstract

The transfer of microorganisms via contaminated small droplets presents a vital pathway to the spread of disease. These droplets can be expelled actively by coughing and sneezing, but also via a proxy, such as splashes from contaminated sinks and toilets. Antimicrobial materials provide a preventative approach to controlling the microbial populations on surfaces to combat this issue. However, currently, there are limited standardized testing methods for assessing the antimicrobial efficacy of surfaces using small droplets in a realistic manner. Furthermore, the impact of moisture is often overlooked in current standards. Thus, a method of assessing antimicrobial materials in realistic conditions using contaminated small droplets was developed to assess the efficacy of a given material under both wetted and dry conditions. Additionally, this method was translated to determine the impact of moisture on the antimicrobial efficacy of copper surfaces against bacteria (methicillin-resistant *Staphylococcus aureus* [MRSA] and *Pseudomonas aeruginosa*), yeast (*Candida albicans*), and viruses (bacteriophage: Φ6 and MS2). For each microorganism, stainless steel (inert) and copper (antimicrobial) surfaces were inoculated with ten 1 µL droplets of microbial inoculum and recovered at the following intervals: immediately, at the time of evaporation of the droplets on the surface (activity when droplet/moisture present), and 2 hours post-evaporation (activity when droplet/moisture absent). Reduced survival of both *P. aeruginosa* and Φ6 phage was observed on stainless steel, indicating they are inappropriate for this method, likely because of desiccation. Copper was demonstrated to have a reduced antimicrobial efficacy under lower relative humidity conditions in all cases compared to reports in the literature, highlighting the benefit of a method that assesses efficacy using approaches more aligned to real-world application of these materials, demonstrating a need for careful consideration regarding the implementation of antimicrobial materials based on methods that might not simulate end-use.

**IMPORTANCE:**

Small droplet transfer represents a key pathway for microbial movement throughout the built environment, contributing to increased healthcare costs and mortality rates, particularly via healthcare-associated infections. Antimicrobial materials can provide a vital passive and low-maintenance method of microbial control within a wider infection control system. However, current standards often use either environmental conditions unrealistic to the end-use environment (e.g., relative humidity above 90% that can overemphasize the efficacy of some materials) and/or methodological specifications that increase operator variability (e.g., manual spreading of inoculum on the surface). This paper describes a method of assessing the antimicrobial efficacy of a surface under both moist and dry conditions via small droplet contamination. The method was applied to antimicrobial copper surfaces that can pass current standards, such as ISO 22196, due to constant moisture availability throughout the test and found a decreased antibacterial and antifungal efficacy while still providing high antiviral efficacy.

## INTRODUCTION

The transmission of microorganisms via surfaces represents a key pathway to the spread of disease throughout the built environment (1). Surfaces can become contaminated with pathogens via several methods, including small droplet inocula (e.g., from coughing and/or sneezing [2]), and act as microbial reservoirs to transfer microorganisms between people (3). This is of particular concern in areas with a high volume of people, such as office and residential buildings (4), healthcare facilities (5), and public transport (6). Expelling droplets by breathing, coughing, and sneezing has been shown to be highly efficient at depositing microorganisms on surfaces that can then result in disease transmission (7).

Within the built environment, the survival of microorganisms on inert surfaces can range from days to weeks, depending on the material type and environmental conditions the microorganisms are subjected to. Typically, however, gram-negative bacteria, fungi, and enveloped viruses (e.g., SARS-CoV-2) are more susceptible to low relative humidity environments and desiccation as compared to gram-positive bacteria and non-enveloped viruses (e.g., norovirus) (8–10). For example, methicillin-resistant *Staphylococcus aureus* (MRSA) has been shown to remain viable for over 6 months (11), while *Pseudomonas aeruginosa* had a large reduction in culturable cells after just 24 hours (12). However, some exceptions exist, such as *Escherichia coli,* that has been found to survive between 14 and 60 days on stainless steel surfaces and 1–300 days on plastic surfaces (13). Within healthcare environments, indirect transfer of microorganisms contributes to secondary healthcare-associated infections (HCAI) that increase healthcare costs, time to recovery, morbidity, and mortality (14, 15).

Antimicrobial materials (AMM) have gained popularity as an additional method of infection control in the built environment (16), reportedly reducing the prevalence of HCAI (17), as well as reducing the burden currently placed on traditional methods, such as antibiotics, that are becoming less effective with the increase in antibiotic resistance (18). Such materials have been used successfully throughout healthcare facilities to help reduce the spread of disease while requiring little maintenance (19). Antimicrobial materials can have a range of mechanisms of action, but generally align with three categories: antibiotic release mechanisms, contact killing, and non-adhesive surfaces (20). The most common of these are contact killing surfaces that include metal-ion release, such as copper and silver incorporated materials (21), photocatalytic materials, such as titanium dioxide and bismuth oxide (22–24), and graphene-based materials (25).

However, prior to the implementation of any new AMM, it is essential to adequately assess the antimicrobial efficacy of a prospective AMM to ensure effectiveness once in use. Standardized test methods allow users to test prospective materials using a method that has undergone rigorous validation, providing confidence that the material will be active when in use (26). However, many current standardized test methods, such as the commonly used ISO 22196 (27), utilize unrealistic environmental conditions that do not reflect end-use scenarios, presenting a possibility for disparity between laboratory results and in-use antimicrobial efficacy. While many aspects of the method can be considered unrealistic (e.g., temperature of 35°C, no airflow present), larger issues arise from the specification that relative humidity (RH) be raised to above 90%. Under these conditions, the inoculum will not evaporate on the surface for the duration of the test (28), allowing materials that require moisture to be active (e.g., copper [29] and silver [30] based surfaces) to have artificially inflated efficacy that will not be exhibited once in use.

Recent advancements have been made in an attempt to improve these issues, with a newly published standard, ISO 7581 (31). This method investigates the antimicrobial efficacy of surfaces under non-submerged conditions, whereby a 1 mL droplet of microbial suspension is transferred to a surface and spread using the pipette tip until dry (usually 3–10 minutes). The surfaces are then incubated at a moderate RH (35%–60%) for 1 to 24 hours prior to recovery. Compared to ISO 22196, this method does offer environmental conditions more analogous to expected end-use scenarios for a wide range of environments. However, the method still introduces variability via spreading the inoculum with the pipette tip, which can be vastly different between individuals and between laboratories (32).

As newer standards move to more realistic environmental conditions, the contact angle of the droplet on the surface should be considered as an important parameter. As a higher contact angle will create a more spherical droplet shape on the surface, the evaporation time of the droplet will increase (due to a lower surface area: volume ratio), and the area of the droplet in contact with the surface will be reduced (33). This will have an impact on surfaces that require moisture to be active and should be considered when performing more realistic testing of antimicrobial surfaces. The impacts of contact angle and evaporation mechanics on surface efficacy and microorganism survival are highly complex, with some previous studies having been performed (34–36). However, a deeper understanding of these interactions would greatly benefit the development of future standards.

To accurately assess the performance of antimicrobial metal-based materials such as copper, it is important to simulate the contamination event and environmental conditions that most closely relate to the end-use conditions. However, as the environmental conditions will differ between countries (37), regions (38), and even buildings (39), it is difficult to develop a single method that will encompass every possible scenario. Therefore, assessing the antimicrobial efficacy of copper surfaces under a range of environmental conditions and during periods of moisture (when the droplet is present on the surface), as well as periods of no moisture (when the droplet has fully evaporated), can provide a more comprehensive overview of a given material’s antimicrobial efficacy once in use.

The aim of this study was to develop a method of accurately and reproducibly assessing the antimicrobial efficacy of copper surfaces under conditions analogous to a small droplet contamination event in a range of RH environments. This method was then applied to a range of microorganisms (*S. aureus*, *P. aeruginosa*, *C. albicans*, Φ6 bacteriophage, MS2 bacteriophage) to assess the full antimicrobial range of copper surfaces incubated under low (<20%), medium (40%–60%), and high (>70%) RH. Furthermore, the contact angles of the contaminated droplets on copper surfaces were examined to determine whether there was any impact on antimicrobial efficacy.

## MATERIALS AND METHODS

### Preparation of materials, solutions, and microbial suspensions

Samples of stainless steel 316L (RS components) and EN1652 grade Cu-ETP copper (minimum 99.9%; RS components) of size 20 × 20 mm were sterilized by wiping thoroughly with a cloth soaked in 70% ethanol, then submerged in 70% ethanol for 30 seconds and dried in a sterile environment for a minimum of 18 hours. A validated recovery medium (SCDLP neutralizer, validation available in Supplementary Information A) was prepared by adding 30 g/L tryptone soya broth (TSB, Fischer Scientific, UK), 1 g/L L-α-Lecithin (Fischer Scientific, UK), and 7 g/L polysorbate 80 (Fischer Scientific, UK) to distilled water, followed by autoclaving at 121°C for 20 minutes. Tryptone soya broth and tryptone soya agar (TSA, Fischer Scientific, UK) were prepared as per the manufacturer’s instructions. Bovine serum albumin (BSA, Fischer Scientific, UK) solution (0.15%) was formed by adding 0.75 g BSA to 500 mL distilled water and filter sterilizing with a 0.2 µm filter (Sarstedt, Germany).

SM buffer was prepared by adding 2 g/L magnesium sulfate heptahydrate (Fischer Scientific, UK) and 5.8 g/L sodium chloride (Fischer Scientific, UK) to 800 mL of distilled water. Separately, a tris-HCl solution was created by adding 12.11 g of tris base (Fischer Scientific, UK) to 80 mL of distilled water and dissolving using a magnetic hotplate stirrer (SLS, UK), then adding 30% hydrochloric acid (Fischer Scientific, UK) until a pH of 7.4–7.6 was achieved. Distilled water was added until a total volume of 100 mL was achieved, then 50 mL of the tris-HCl solution was added to the 800 mL solution, which was then adjusted to 1 L with distilled water. The SM buffer was then separated into 500 mL aliquots and autoclaved at 121°C for 20 minutes. Malt Extract Agar (MEA; SLS, UK) and Sabouraud Dextrose Broth (SAB broth; Fischer Scientific, UK) were prepared according to the manufacturer’s instructions and autoclaved at 121°C for 20 minutes.

Suspensions of bacteria and yeast (*Pseudomonas aeruginosa* NCTC 8060/methicillin-resistant *Staphylococcus aureus* NCTC 13143/*Candida albicans* ATCC 10231) were formed by adding one colony of either bacterial strain from a streak plate on TSA to 10 mL TSB or one yeast colony from an MEA streak plate to 10 mL SAB broth and incubated at 37°C for 18–24 hours, shaking at 150 rpm in an orbital incubator (Sciquip, UK) with a throw of 40 mm. The cultures were then centrifuged at 3,500 rpm (2,342 × *g*) for 10 minutes (bacteria) or 3,000 rpm (1,721 × *g*) for 5 minutes (yeast) in a Sigma 3-16 L centrifuge (Sigma, UK), the supernatant removed and 10 mL 0.15% BSA solution added, followed by vortex mixing (Clifton, UK) to homogenize the suspension. The process of centrifugation to homogenization was repeated once more to ensure minimal nutrients in the final working culture. The optical density of the suspension was adjusted to 0.5 ± 0.005 at 600 nm using a spectrophotometer (Jenway 6305, UK), adding 0.15% BSA solution where required. The bacterial suspensions were serially diluted (1:9) twice in BSA solution to achieve a final concentration of ~2–7 × 10^6^ cells/mL. As a cell suspension of *C. albicans* at 0.5 OD at 600 nm forms a concentration of ~2–7 × 10^6^ cells/mL, no dilution was required. However, the concentration was confirmed by cell counting (hemocytometer: ^1^/_400_ mm^2^ × 0.1).

Bacteriophage suspensions were created by performing a plaque assay from a previous stock to generate plaques. Briefly, the concentrated phage suspension was diluted 1:9 in SM buffer to 10^−8^, then 100 µL of each phage dilution was mixed in a 5 mL phage tube (Simport scientific, Canada) with 300 µL of appropriate host in triplicate (*Pseudomonas syringae* ATCC 21781 for Φ6 ATCC 21781-B1, *Escherichia coli* ATCC 12435 for MS2 ATCC 15597-B1) and allowed to mix for 8 minutes before adding 3 mL ½ concentration molten TSA heated to 50°C in a water bath (Grant instruments, UK) immediately after autoclaving and pouring rapidly on to a TSA plate while circulating the agar plate to ensure even coverage. Plates were then incubated inverted at 30°C (*P. syringae*) or 37°C (*E. coli*) for 24 hours. Plates with >500 plaques were then used for phage harvesting, whereby 5 mL SM buffer was added to the agar plate for 1 hour to allow the phage to diffuse out, then the SM buffer was poured into a universal tube (Fisher Scientific, UK) and filter sterilized (0.2 µm filter). The plaque assay was then run in the same manner once more to confirm the concentration of phage in the suspension, which was then diluted appropriately in 0.15% BSA solution to achieve a final concentration of 2–7 × 10^6^ PFU/mL.

Three chambers (polypropylene boxes) of size 260 × 260 × 170 mm had saturated salts (200 g with 50 mL distilled water) added to the bottom to adjust the RH to either low (<20%, lithium chloride, Fischer Scientific, UK), medium (40%–60%, potassium carbonate, Fischer Scientific, UK) or high (>70%, sodium chloride, Fischer Scientific, UK). Each chamber was sealed and allowed to equilibrate for a minimum of 24 hours before use.

### Contact angle analysis of droplets

Contact angles of 1 µL droplets of each microorganism on copper and stainless-steel surfaces were determined. Each microorganism was prepared, and 1 µL was pipetted onto each surface. Immediately after, a goniometer (Kruss MobileDrop GH11) was placed over the droplet, and the tangent contact angle was measured. Each microorganism was measured on five separate occurrences (with five separate droplets), and a non-contaminated droplet (BSA solution only) was also measured as a control. To determine statistical significance, an ANOVA was performed with a post hoc Tukey test.

### Antimicrobial assessment of copper surfaces

Stainless steel and copper coupons were separated into triplicate in sterile petri dishes, followed by pipetting ten 1 µL droplets onto each coupon (18 coupons total; nine stainless steel/nine copper). Four petri dishes (12 coupons total, six stainless steel, six copper) were placed into a chamber (either low, medium, or high RH), the lids of the petri dish removed, and a temperature/relative humidity sensor (Extech, UK) placed in the chamber, the chamber was then sealed and placed in an incubator set to 20°C. A timer was immediately started, and the remaining six coupons were recovered into a 5 mL SCDLP neutralizer in an 80 mL stomacher bag. The coupons were manually agitated for 30 ± 5 seconds, and the SCDLP was transferred to a 30 mL universal tube, followed by vortexing for 15 seconds to homogenize. An aliquot of 200 µL was then transferred in triplicate to a 96-well plate and serially diluted 1:9 (20:180 µL) to 10^−2^. Spot plating (10 µL) of each dilution was performed on a large bioassay dish (125 × 125 mm; Fischer Scientific, UK) of TSA. Additionally, 100 µL of the undiluted recovery medium was spread plated onto TSA agar plates. Once the droplets had evaporated on the coupons (time T), two more petri dishes (three stainless steel coupons, three copper coupons) were recovered, and the same process was repeated. The evaporation time was recorded for each case. At 2 hours post-evaporation (T+2 h), the same process was repeated for the remaining coupons. All plates were then incubated at 37°C for 24 hours, and colony-forming units (CFU) were counted.

The same process was repeated for the bacteriophage suspensions to determine antiviral activity, except for the following changes. After recovery of the coupons into SCDLP, serial dilution occurred in 30 mL universal tubes in SM buffer 1:9 (1 mL:9 mL) to 10^−2^ and 100 µL of each dilution was pipetted in triplicate into 5 mL phage tubes (SLS, UK) alongside 300 µL of host bacteria. After allowing to mix for 8–10 minutes, 3 mL of ½ concentration TSA (20 g/L) was added to each phage tube, the tube was mixed by rubbing hands together around the tube for 5 seconds, and poured onto a TSA agar plate evenly, allowed to dry, and incubated inverted at the temperature appropriate for the host bacteria.

### Comparison to current standard: ISO 22196

In addition, ISO 22196 was also performed to compare the novel method to a currently available standard that utilizes a full and continuous wet deposition and incubation. The methodology used following ISO 22196 is as follows:

If used, all materials and solutions were prepared as stated above. Saline solution was prepared by adding 0.85% wt/vol sodium chloride to deionized water. Additionally, 1/500 nutrient broth was prepared by aliquoting 1 mL of nutrient broth (prepared as per the manufacturer’s instructions) to 499 mL of deionized water. Both were sterilized by autoclaving at 121°C for 20 minutes. An inoculum of either *Staphylococcus aureus, Pseudomonas aeruginosa,* or *Candida albicans* was prepared by transferring one colony from a TSA (bacteria) or MEA (yeast) plate to perform a streak plate and incubating at 37°C for 18–24 hours. Colonies from this plate were transferred to 10 mL 1/500 nutrient broth to achieve an optical density of 0.5 ± 0.005 OD at 600 nm. For the bacterial cultures, the inoculum was then diluted 1:9 three times in 1/500 nutrient broth to create the working culture at a final cell concentration of ~2–7 × 10^5^ cells/mL. Six stainless steel and three copper samples were inoculated with 100 µL of the working culture and covered with a polypropylene square of size 18 × 18 mm, sterilized in 70% ethanol for 30 seconds, and allowed to dry overnight. Three stainless steel coupons were immediately recovered into a 5 mL neutralizer, and the total viable count was determined in the same manner as previously described. The remaining three stainless steel and three copper coupons were transferred to a chamber (size 300 × 300 × 175 mm) set to >90% relative humidity (by adding deionized water such that the bottom 5 mm of the chamber was submerged) and a grid added to allow samples within petri dishes to be placed on top while still allowing humidification of the air. After 24 hours of incubation in the chamber, the recovery and plating process was repeated in the same manner for the remaining six coupons. The same process was repeated for both bacteriophage, performing plaque assays as previously described.

### Statistical analysis and data visualization

Statistical analyses were performed, and graphs were produced using GraphPad Prism (version 9.5.1). For all antibacterial efficacy data, normality was verified using a Shapiro-Wilk test, and a Kruskal-Wallis test was run. If the result was significant, pairwise Wilcoxon rank tests between conditions were completed to statistically compare the results. A *P* value of *P* < 0.05 was considered significant.

## RESULTS

### Contact angle analysis of droplets

**Fig 1:**
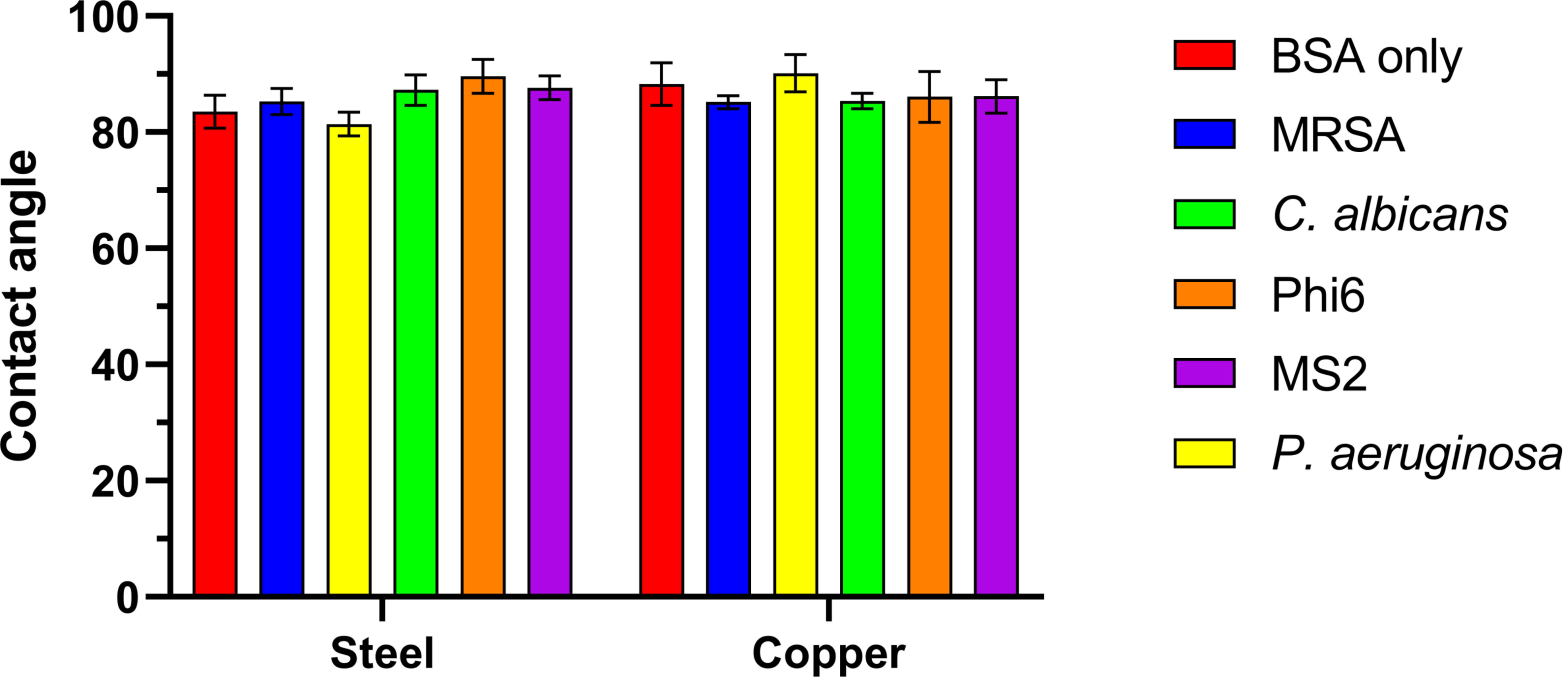
Contact angles generated from 1 μL droplets of 0.15% BSA, *S. aureus* NCTC 13143 (MRSA), *P. aeruginosa* NCTC 8060, *C. albicans* ATCC 10231, Φ6 ATCC 21781-B1, and MS2 ATCC 15597-B1, measured immediately after transfer by pipette to stainless steel and copper surfaces using a Kruss MobileDrop GH11.

The contact angles for each suspension are shown in Fig. 1. All contact angles were between 80° and 90° on both materials. Droplets containing bacteria on stainless steel have lower contact angles than those containing fungi and bacteriophage, with contact angles of droplets of *P. aeruginosa* being statistically significantly lower than droplets containing *C. albicans* (*P* = 0.0218), Φ6 (*P* = 0.0003), and MS2 (0.0113). No statistical significance was observed on copper coupons.

### Methodological proof of principle: antimicrobial assessment of copper

#### Evaporation time

**Fig 2:**
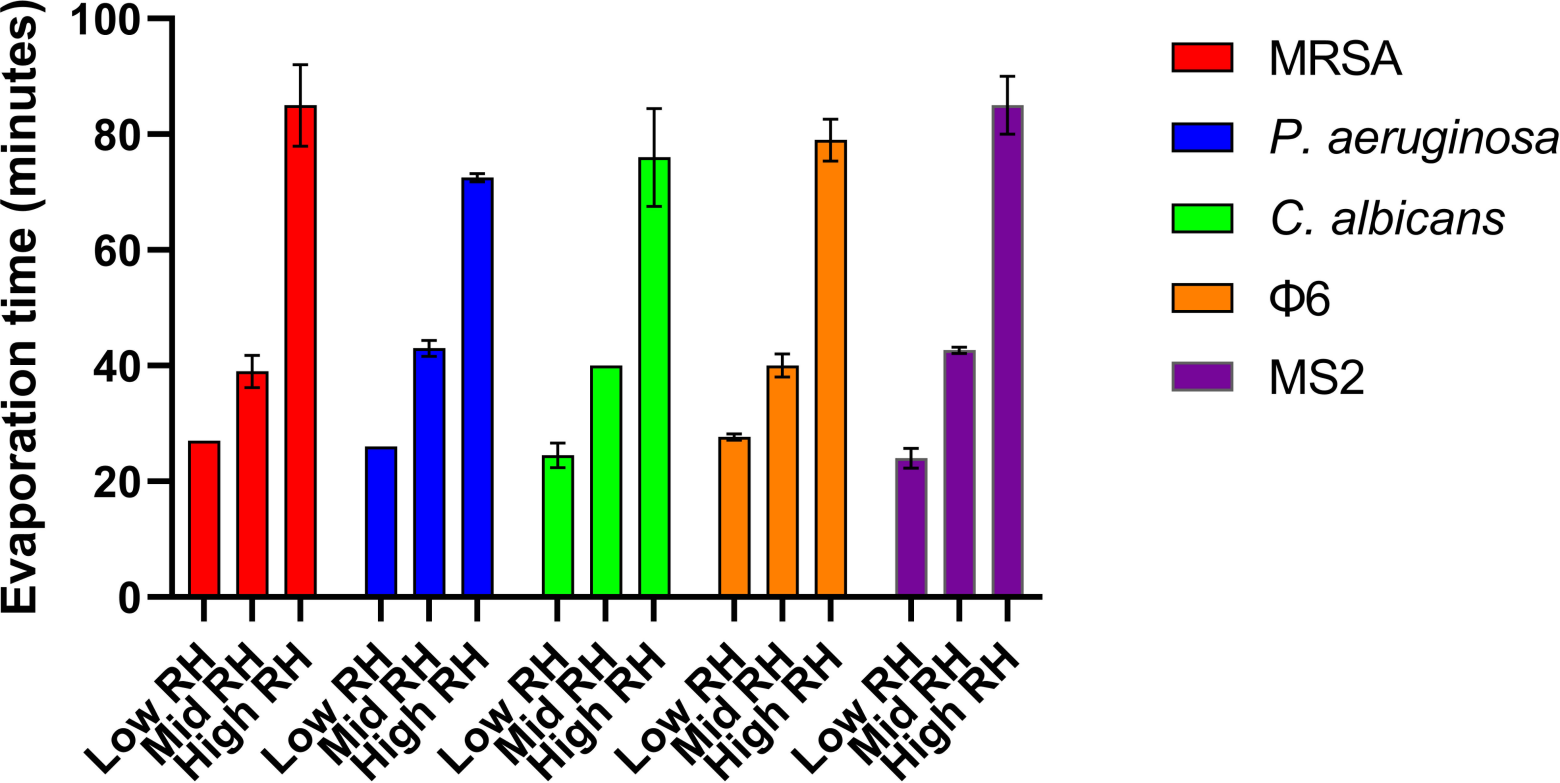
Average evaporation times for ten 1 μL droplets of *S. aureus* NCTC 13143 (MRSA), *P. aeruginosa* NCTC 8060, *C. albicans* ATCC 10231, Φ6 ATCC 21781-B1, and MS2 ATCC 15597-B1 on stainless steel and copper surfaces when exposed to low (<20%), medium (40%–60%), or high (>70%) relative humidity environments.

The evaporation time of the droplets was measured during each test (Fig. 2), where general consistency was observed at both low (range: 23–26 minutes) and mid (range: 37–44 minutes) RH environments. A larger variation in evaporation time was observed in the high RH environments, ranging from 70 to 90 minutes.

### Antimicrobial activity of copper coupons—bacteria and yeast

**Fig 3:**
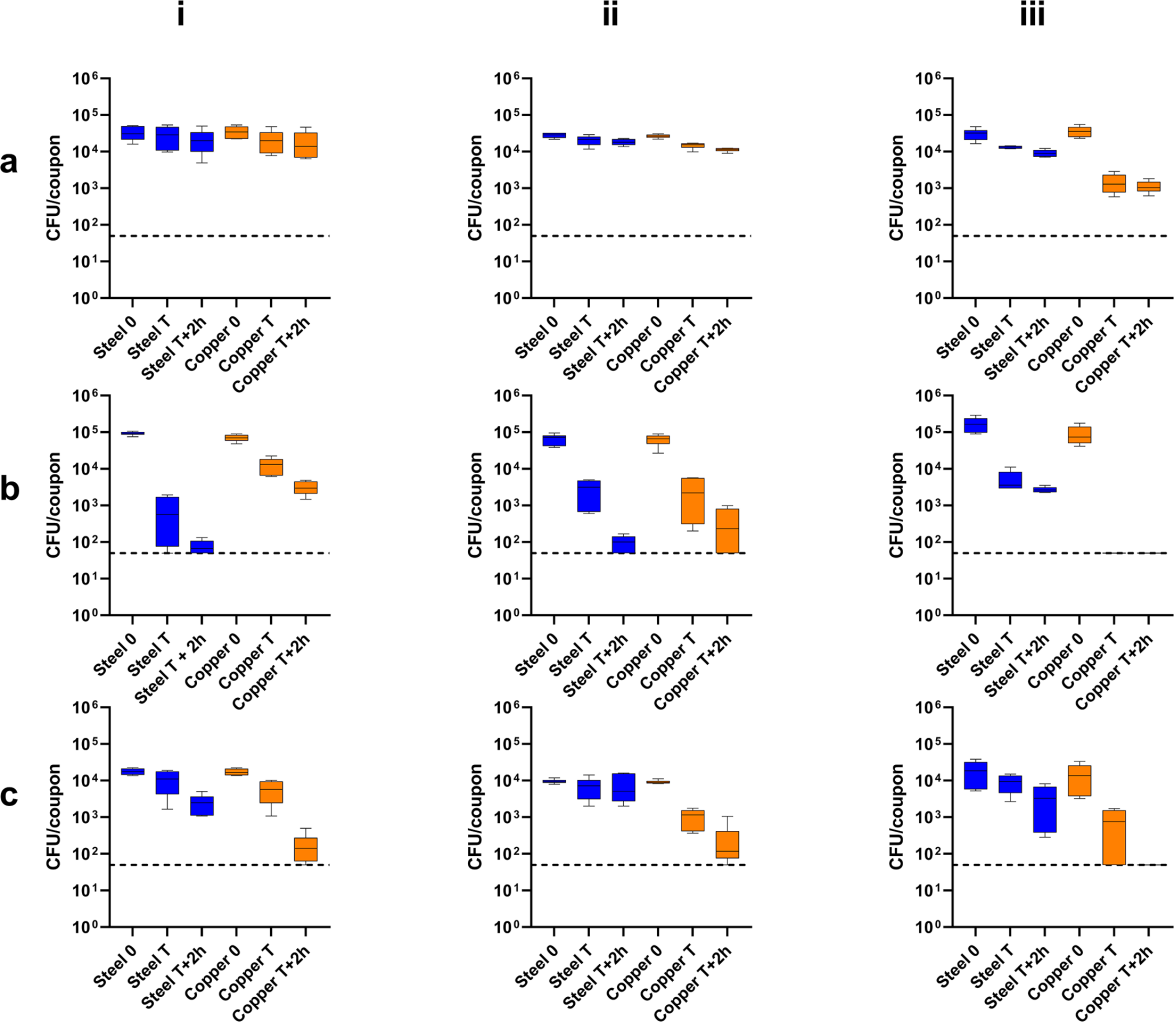
Colony-forming unit counts from an inoculum of (**a**) *Staphylococcus aureus* NCTC 13143, (**b**) *Pseudomonas aeruginosa* NCTC 8060, or (**c**) *Candida albicans* ATCC 10231 applied as ten 1 µL droplets to steel (blue) and copper (orange) coupons. The time of recovery was immediately (0), the time of evaporation of the droplets on the surface (T), and 2 hours post-evaporation of the inoculum (T+2 h). The red dots and black dotted line represent the individual values and the limit of detection, respectively. Surfaces were incubated at room temperature and either (i) <20%, (ii) 40%–60%, or (iii) >70% relative humidity.

The populations recovered from stainless steel for MRSA were consistent throughout the test, with statistical significance only observed in a 0.2-log decrease between the populations at time zero and time of evaporation (*P* < 0.0001) in the high RH conditions (Fig. 3a, iii). Conversely, populations recovered from stainless steel for *P. aeruginosa* and *C. albicans* were significantly lower at the time point T+2 h in most RH conditions, with reductions between 1.9 and 3.2 log for *P. aeruginosa* (*P* < 0.0001 in all cases) and a 0.9 and 1.2 log reduction for *C. albicans* (*P* < 0.0001), whereas no significance was observed in mid RH conditions (reduction: 0.2 log, *P* = 0.5467).

Greater reductions in populations recovered from copper coupons were observed for MRSA. However, this was minimal and not statistically significant for the low (*P* = 0.5178) RH environments. Statistically significant decreases were observed in both medium (*P* < 0.0001) and high (*P* < 0.0001) RH environments, with a much greater absolute reduction (~1.25 log) in a high RH environment. Similar increases in population reduction were observed for *C. albicans*, where a reduction between 1.6 log (low RH) and complete reduction (high RH) was observed (*P* < 0.0001 for all cases). While reductions in populations were observed for *P. aeruginosa*, the reductions were lower than from stainless steel for low RH (1.4 log, *P* < 0.0001) and mid RH (1.3 log, *P* < 0.0001) conditions. A complete reduction was observed in the high RH conditions (*P* < 0.0001).

### Antimicrobial activity of copper coupons—bacteriophage

**Fig 4:**
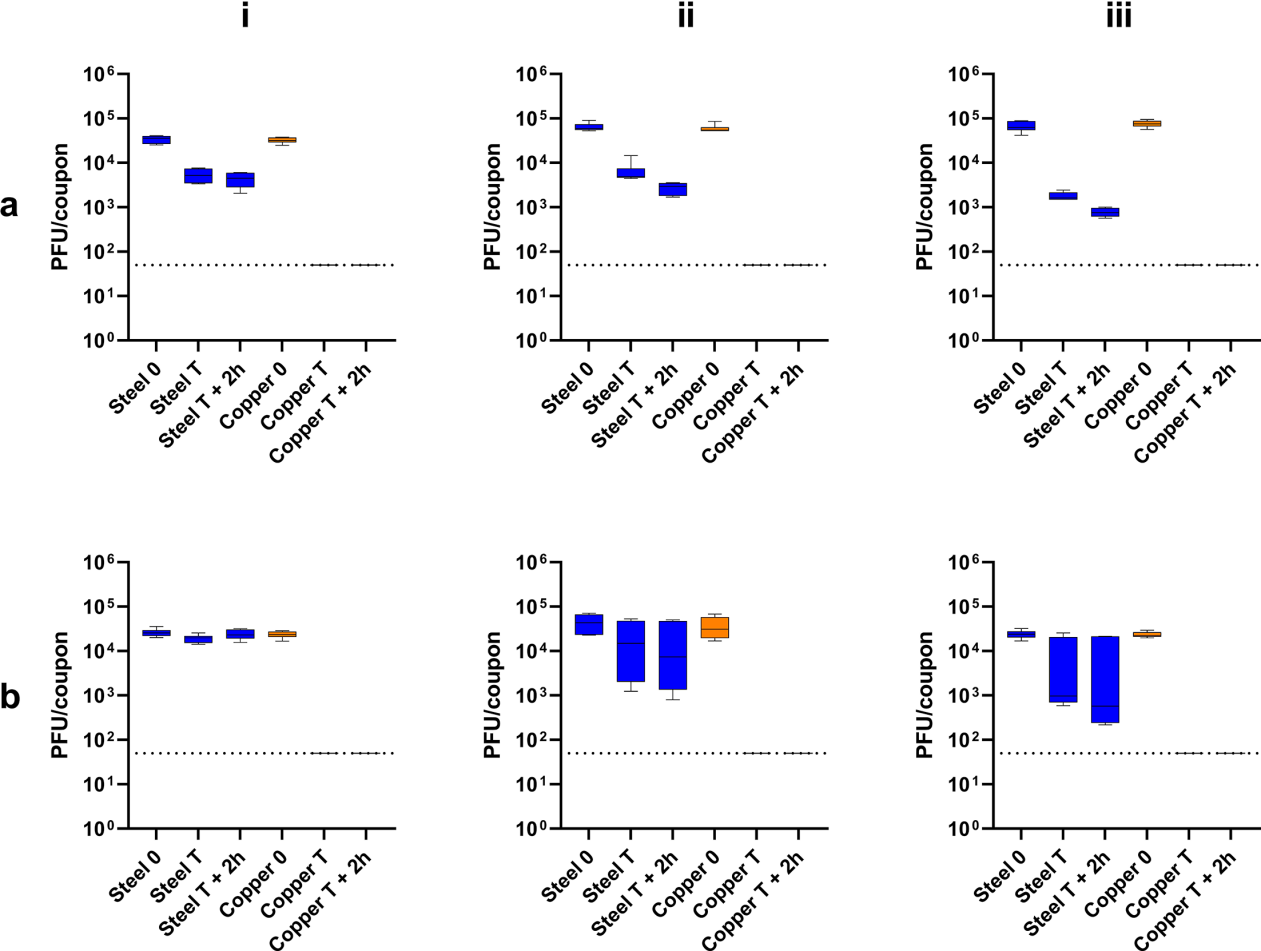
Plaque-forming unit counts from an inoculum of (**a**) Φ6 bacteriophage ATCC 21781-B1 or (**b**) MS2 bacteriophage ATCC 15597-B1 applied as 10 × 1 µL droplets to steel (blue) and copper (orange) coupons. The time of recovery was immediately (0), the time of evaporation of the droplets on the surface (T), and 2 hours post-evaporation of the inoculum (T+2 h). The red dots and black dotted line represent the individual values and the limit of detection, respectively. Surfaces were incubated at room temperature and either (i) <20%, (ii) 40%–60%, or (iii) >70% relative humidity.

The populations recovered from stainless steel were statistically significantly reduced in all RH environments for Φ6 bacteriophage (low: 0.9 log, mid: 1.4 log, high: 1.9 log, all *P* values < 0.0001), only the high RH environment reduced the MS2 bacteriophage populations significantly (*P* < 0.01). On copper coupons, a statistically significant (*P* < 0.0001) and complete reduction was observed in all RH conditions for both Φ6 and MS2 bacteriophage (Fig. 4).

### Comparison to current standard: ISO 22196

**Fig 5:**
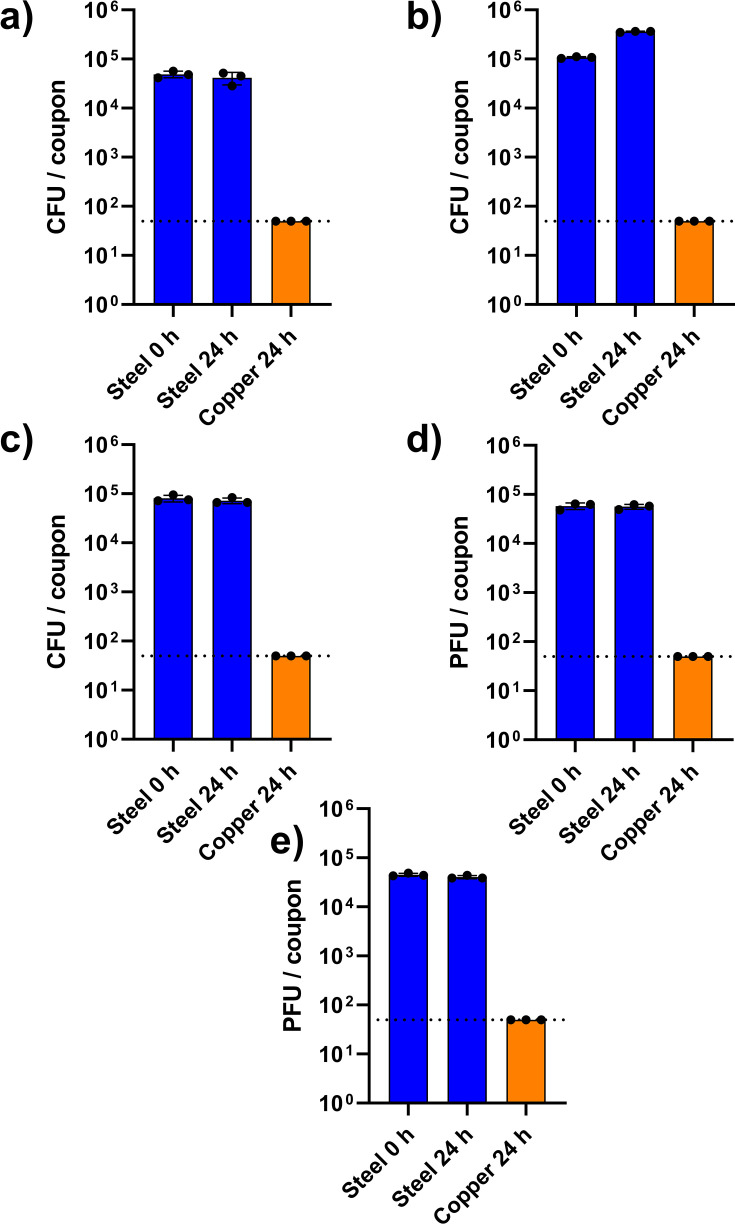
Survival of (**a**) MRSA, (**b**) *P. aeruginosa*, (**c**) *C. albicans*, (**d**) Φ6, and (**e**) MS2 on stainless steel and copper surfaces when populations were recovered immediately and after 24 hours when following ISO 22196 methodology. (•) represents the arithmetic means of three technical repetitions (repeats from a single coupon/neutralizer solution). Dashed line represents the limit of detection (5 × 10^1^ CFU/cm^2^).

When assessing antimicrobial efficacy according to ISO 22196 (Fig. 5), a statistically significant reduction (*P* < 0.0001) in the populations of all microorganisms recovered from copper surfaces to the limit of detection was observed after 24 hours (*P* < 0.0001 in all cases). Also, as expected, statistically similar populations were recovered from stainless steel coupons at 0 and 24 hours for all microorganisms (MRSA: *P* = 0.5588, *P. aeruginosa*: *P* = 0.1392, *C. albicans*: *P* = 0.5408, Φ6: *P* = 0.9366, MS2: *P* = 0.1119).

## DISCUSSION

The aim of this study was to develop a method for antimicrobial efficacy analysis of surfaces contaminated via small droplets that accounts for a range of relative humidity environments. This method was then applied to copper surfaces to determine the impact of moisture on the antimicrobial efficacy of copper surfaces against a range of microorganisms, whereby removing available moisture (e.g., with lower RH) reduced the overall efficacy in most cases. Additionally, physical factors, such as the contact angles of the droplets on the surface, were measured to assess any potential impact on overall efficacy.

There are a range of methods both in the literature and as standard methods that aim to assess the antimicrobial efficacy of non-porous materials using a liquid inoculum. Established standardized test methods (e.g., ISO 22196 [27] and ASTM E2180 [40]) tend to favor reproducibility and ease of testing over being entirely representative of an end-use scenario and may overemphasize the efficacy of some materials, particularly if it requires moisture to exhibit antimicrobial properties (e.g., materials incorporating metal ion release mechanisms). This can be seen clearly when comparing the data generated between the novel method and ISO 22196 in this study, where complete kill is observed in every case when performing ISO 22196. More recently, there has been development toward a more realistic standardized methodology via ISO 7581 (31) that uses small volume droplets similar to that described in this study, but with manual spreading of the inoculum on the surface that may increase variability between operators and differing time points for surface recovery. The method described here offers a complementary methodology that aims to determine the effects of relative humidity and transient moisture on surface efficacy. Within the literature, several studies have assessed the antimicrobial efficacy of copper surfaces under environmental conditions more analogous to end-use environments. For example, one study found rapid (5–60 minutes) death (particularly in mutants) of *C. albicans* cells after deposition using a swab and incubation at 23°C (41). Small droplets (1–25 µL) have also been used to test copper surfaces under more realistic conditions (e.g., room temperature) in several studies, demonstrating inactivation of MRSA in 10–90 minutes (42, 43), *E. coli* in 10 minutes (44), *P. aeruginosa* in 240 minutes (45), Phi6 in 15 minutes (46), and MS2 in 5 minutes (47). However, the RH was rarely considered throughout the literature, rather opting for ambient RH that can widely vary depending on a range of factors (time of year, location, etc). The slower antimicrobial action of the copper surfaces in this study compared to the literature may be explained by the lower inoculum volume applied to the surface and allowing the droplets on the surface to fully evaporate.

As expected, the antimicrobial efficacy of copper varies between microorganisms, likely due to differing modes of action and cell structure or physiology. However, other factors can also affect the suitability of a microorganism for an antimicrobial efficacy test method. For example, as the populations of *P. aeruginosa* (gram-negative bacteria) and Φ6 phage (enveloped virus) can also decline on the inert surface (stainless steel) due to desiccation (48, 49), making efficacy assessment inherently challenging, exacerbated by both lower RH and longer contact time in high RH environments.

Generally, gram-positive bacteria have a much higher resistance to desiccation than gram-negative bacteria (50), similarly with non-enveloped (e.g., MS2) to enveloped (e.g., Φ6) viruses, respectively (51), although the reasons underpinning this are poorly understood. Links between the bacterial cell wall width and peptidoglycan production in response to low water activity conditions, as well as the production of water stress proteins (that enhance rehydration capabilities) in *S. aureus,* have been demonstrated (52, 53), that could explain the increased survival of gram-positive bacteria compared to gram-negative (that lack a thick outer peptidoglycan cell wall layer). Additionally, yeast generally has low desiccation resistance, and growth and survival are greater in higher RH conditions (41), alongside a reduced capacity for fungi to germinate (54). Overall, however, as the desiccant-sensitive microorganisms would naturally have reduced survival in end-use scenarios that experience lower RH, the benefit of adding antimicrobial functionality to a material specifically for these circumstances would be limited.

While RH can directly impact the survival of microorganisms due to desiccation, it can also affect the potential antimicrobial efficacy of copper surfaces, as available moisture is a key requirement for the release, dispersion, and microbial uptake of copper ions. This highlights the potential complexity of incorporating RH into a wide range of antimicrobial efficacy standards and the requirement for careful consideration and planning in these processes. Generally, the antimicrobial efficacy of copper surfaces appears to be positively correlated with RH and moisture present on the surface (48) and is demonstrated both across the RH ranges in the novel method, as well as when comparing the high antimicrobial efficacy in ISO 22196 to the more limited efficacy observed in the novel method. Overall, the antiviral efficacy of copper far exceeds the antibacterial or antifungal efficacy, and as such, could be specialized for that purpose when under “harsher” environmental conditions (55). This may be explained by the varying mechanisms of action of copper against different classes of organism (bacteria/yeast/viruses) (56). As viruses are generally much simpler and have no defense mechanisms against antiviral treatments when outside the host, a faster antimicrobial efficacy is to be expected, whereas bacteria and fungi may employ efflux pumps for copper ions or DNA damage protection and repair mechanisms to mitigate the effects of the copper on the cells (57). Overall, the current methodology does not consider the effects of RH on the antimicrobial efficacy of the material, which can then allow materials that would not provide efficacy in end use to be approved. However, more recently, RH has been recognized as an important factor for antimicrobial material efficacy that should be reflected in the testing methodology. For example, using a 5 × 2 µL inoculum size, copper, silver, and silicone-quat coatings were assessed for antimicrobial efficacy against *S. aureus* and *Escherichia coli* in a similar range of RH values as this study, which also found a reduction in efficacy in lower RH conditions (58).

As the method described here assesses the materials’ efficacy during the periods of present and absent moisture in differing RH environments, the differential time of evaporation of the droplets on the surface may have also provided a generally greater antimicrobial efficacy. The decision to avoid a pre-defined (and potentially arbitrary) time point allows for an accurate simulation of surface antimicrobial efficacy during small droplet contamination of surfaces, whereby frequent interaction is lacking (which would therefore provide the required time for the droplet to fully evaporate). Additionally, if preferred or required, the method could be adapted to use fixed material recovery times (e.g., every 1, 2, 24 hours, etc.) to reflect the cleaning schedule of the end-use case. For example, hospital surfaces, particularly in the ICU, have strict cleaning regimens and require all surfaces to be cleaned at least once per day, with stricter regimens for patient contact surfaces (59).

To best apply this method for international use (i.e., due to differences in environmental conditions between countries), it is of benefit to link RH ranges (as they are most closely linked to efficacy in many instances) to a generic group of end-use scenarios (e.g., the indoor built environment). For example, prospective materials could be tested at the three ranges (low: <20%, medium: 40%–60%, high: >70% RH) and only approved for the end-use scenarios of that specific range (e.g., mid RH may relate to indoor acclimatized settings [60]) if the material demonstrates efficacy under those conditions, allowing for customized approval depending on the large number of factors that can affect a material’s efficacy (e.g., temperature, RH, conditioning films, etc). Additionally, further study of the factors affecting antimicrobial surface efficacy and microorganism survival on inert surfaces would provide further confidence in the accuracy and reproducibility of the method across the range of potential microorganisms and environmental conditions that could be employed. For example, the rate of decay of Φ6 phage is dependent on the initial concentration added to the surface (61). This more detailed approach would also allow materials that only provide antimicrobial efficacy in high RH conditions (e.g., silver-based materials) to avoid an outright ban in use when they may still provide benefit in some key areas, such as in catheters, where available moisture is high, while limiting overuse in lower RH areas where the benefits would be lacking, such as on door handles.

### Conclusion

The use of antimicrobial materials in a wide range of applications heavily relies on a standardized testing method that accurately and reproducibly simulates the environment that the prospective material will be applied to. The method described demonstrates reproducible assessment of antimicrobial surfaces contaminated via small droplet inocula (e.g., via sneezing) that can complement current standards and specifically highlights the impact of relative humidity on the antimicrobial efficacy of moisture. The work presented here provides a protocol that can be applied to a wide range of microorganisms and environments that would allow for a comprehensive understanding of a material’s potential use and where efficacy may be lacking. The method can facilitate standardization of efficacy assessment between materials that simulate end-use environments and ease comparison, while preventing exacerbation of resistance through overuse of antimicrobials. Additionally, this work facilitates the development of either a novel standard or a variant to ISO 7581 to provide a more comprehensive analysis of antimicrobial material efficacy.

### Article highlights

- Development and validation of a method to assess antimicrobial materials contaminated by small droplets under realistic conditions.
- The antibacterial and antifungal efficacy of copper coupons is reduced when exposed to lower relative humidity environments.
- High antiviral efficacy of copper was observed in all relative humidity environments.
- Lower (more hydrophilic) contact angles were observed for non-contaminated and bacteria-contaminated droplets on stainless steel.

## Data Availability

Data supporting this publication are openly available from Manchester Metropolitan University’s research repository at https://doi.org/10.23634/MMU.00644221.

## SUPPLEMENTAL MATERIAL

File S1A aem.02304-25-s0001.docx Detail for neutralizer validation.

File S1B aem.02304-25-s0002.xlsx Data supporting neutralizer validation.

Graphical abstract aem.02304-25-s0003.tif Visual depiction of the study.

ASM does not own the copyrights to Supplemental Material that may be linked to, or accessed through, an article. The authors have granted ASM a non-exclusive, world-wide license to publish the Supplemental Material files. Please contact the corresponding author directly for reuse.
