# Supplementary material for: Assessing antibacterial, antiviral, and antifungal efficacy of non-porous materials using small droplet transfer: the simulated splash method
Source: Appl Environ Microbiol. 2026 Apr 1;92(4):e02304-25. doi: 10.1128/aem.02304-25 (PMC13101517; doi:10.1128/aem.02304-25)
Supplement: File S1A — Detail for neutralizer validation. [file aem.02304-25-s0001.docx]

**Supplementary information A**

**Neutraliser validation**

A validation test of the neutraliser (SCDLP) was performed to ensure no toxicity or continued antibacterial efficacy was occurring during the neutralisation period of the studies. The method was adapted from BS EN 1276:2019 (European Committee for Standardisation, 2019) for environmental condition requirement of the novel method.

**Method**

Bacteria and yeast

All materials and solutions were prepared as stated in the manuscript. Ten 1 µL droplets of 0.15 % BSA were transferred by pipette to two copper coupons and incubated in a sterile environment at room temperature and 40 – 60 % relative humidity until two hours post evaporation of the droplets (approximately two hours and thirty minutes). Each coupon was transferred separately to an 80 mL stomacher bag (marked C), with 10 mL SCDLP neutraliser added to each. Additionally, 10 mL sterile water (marked A) and 10 mL neutraliser (marked B) were transferred to 80 mL stomacher bags in duplicate. After 5 minutes, the coupons in stomacher bags C were massaged and a 100 µL inoculum of either *Staphylococcus aureus* NCTC 13143*, Pseudomonas aeruginosa* NCTC 8060 or *Candida albicans* NCTC 12401 at ~5 × 10^4^ cells / mL added to each bag. The inoculum was diluted 1 : 9 to 10^-2^, 100 µL was then spread plated on to TSA in triplicate to confirm the initial cell concentration. Then, 30 minutes post-inoculation, the neutraliser from each bag was transferred to a 30 mL universal tube, homogenised with a vortex mixer and 1 mL for each test added to a sterile empty Petri dish for pour plating with TSA.

Bacteriophage

The method was performed in the same manner as for bacteria and yeast except phage assays were performed on the inoculum to confirm the initial concentration and after the homogenisation step, 100 µL of undiluted neutraliser or distilled water was used to perform phage assays.

**Results**

An acceptable pass rate of for both test comparisons (A-B / A-C) was achieved, providing acceptable counts (30 – 300 colonies present) in all cases. Therefore, the neutraliser can be considered validated for each bacteria, yeast and bacteriophage.

*Staphylococcus aureus*

Table 1. Colony forming unit (CFU) counts recovered from (A) distilled water, (B) neutraliser, and (C) neutraliser with a copper coupon added, after inoculation with Staphylococcus aureus. Additionally, spread plating of the bacterial inoculum was performed to confirm initial cell concentration. All counts were performed from a pure (zero) dilution of the respective samples (pour plate) and a 10^-2^ dilution of inoculum (spread plate). Averages of samples are displayed as per mL.

| Test | Repeat | Colony count | CFU / mL |
| --- | --- | --- | --- |
| Inoculated water (A) | 1 | 468 | 468 |
| Inoculated water (A) | 2 | 471 | 471 |
| Inoculated neutraliser (B) | 1 | 439 | 439 |
| Inoculated neutraliser (B) | 2 | 465 | 465 |
| Inoculated neutraliser with coupon (C) | 1 | 473 | 473 |
| Inoculated neutraliser with coupon (C) | 2 | 472 | 472 |
|  |  |  |  |
| Inoculum spread plates | 1 | 55 | 55000 |
| Inoculum spread plates | 2 | 65 | 65000 |
| Inoculum spread plates | 3 | 45 | 45000 |

| A average | B average | C average |  | A-B recovery rate | 96.27% |
| --- | --- | --- | --- | --- | --- |
| 469.5 | 452 | 472.5 |  | **A-C recovery rate** | 100.64% |

*Pseudomonas aeruginosa*

Table 2. Colony forming unit (CFU) counts recovered from (A) distilled water, (B) neutraliser, and (C) neutraliser with a copper coupon added, after inoculation with Pseudomonas aeruginosa. Additionally, spread plating of the bacterial inoculum was performed to confirm initial cell concentration. All counts were performed from a pure (zero) dilution of the respective samples (pour plate) and a 10^-2^ dilution of inoculum (spread plate). Averages of samples are displayed as per mL.

| Test | Repeat | Colony count | CFU / mL |
| --- | --- | --- | --- |
| Inoculated water (A) | 1 | 430 | 430 |
| Inoculated water (A) | 2 | 461 | 461 |
| Inoculated neutraliser (B) | 1 | 428 | 428 |
| Inoculated neutraliser (B) | 2 | 444 | 444 |
| Inoculated neutraliser with coupon (C) | 1 | 481 | 481 |
| Inoculated neutraliser with coupon (C) | 2 | 467 | 467 |
|  |  |  |  |
| Inoculum spread plates | 1 | 123 | 123000 |
| Inoculum spread plates | 2 | 138 | 138000 |
| Inoculum spread plates | 3 | 121 | 121000 |

| A average | B average | C average |  | A-B recovery rate | 97.87% |
| --- | --- | --- | --- | --- | --- |
| 445.5 | 436 | 474 |  | **A-C recovery rate** | 106.40% |

*Candida albicans*

Table 3. Colony forming unit (CFU) counts recovered from (A) distilled water, (B) neutraliser, and (C) neutraliser with a copper coupon added, after inoculation with Candida albicans. Additionally, spread plating of the bacterial inoculum was performed to confirm initial cell concentration. All counts were performed from a pure (zero) dilution of the respective samples (pour plate) and a 10^-2^ dilution of inoculum (spread plate). Averages of samples are displayed as per mL.

| Test | Repeat | Colony count | CFU / mL |
| --- | --- | --- | --- |
| Inoculated water (A) | 1 | 372 | 372 |
| Inoculated water (A) | 2 | 351 | 351 |
| Inoculated neutraliser (B) | 1 | 352 | 352 |
| Inoculated neutraliser (B) | 2 | 406 | 406 |
| Inoculated neutraliser with coupon (C) | 1 | 378 | 378 |
| Inoculated neutraliser with coupon (C) | 2 | 412 | 412 |
|  |  |  |  |
| Inoculum spread plates | 1 | 54 | 54000 |
| Inoculum spread plates | 2 | 35 | 35000 |
| Inoculum spread plates | 3 | 45 | 45000 |

| A average | B average | C average |  | A-B recovery rate | 106.9% |
| --- | --- | --- | --- | --- | --- |
| 510 | 545 | 525 |  | **A-C recovery rate** | 102.9% |

Φ6 bacteriophage

Table 4. Plaque forming unit (PFU) counts recovered from (A) distilled water, (B) neutraliser, and (C) neutraliser with a copper coupon added, after inoculation with Φ6 bacteriophage. Additionally, phage assays of the bacteriophage inoculum was performed to confirm initial virion concentration. All counts were performed from a pure (zero) dilution of the respective samples and a 10^-2^ dilution of inoculum. Averages of samples are displayed as per mL.

| Test | Repeat | Plaque count | PFU / mL |
| --- | --- | --- | --- |
| Inoculated water (A) | 1 | 46 | 460 |
| Inoculated water (A) | 2 | 56 | 560 |
| Inoculated neutraliser (B) | 1 | 57 | 570 |
| Inoculated neutraliser (B) | 2 | 52 | 520 |
| Inoculated neutraliser with coupon (C) | 1 | 57 | 570 |
| Inoculated neutraliser with coupon (C) | 2 | 48 | 480 |
|  |  |  |  |
| Inoculum spread plates | 1 | 248 | 24800 |
| Inoculum spread plates | 2 | 237 | 23700 |
| Inoculum spread plates | 3 | 271 | 27100 |

| A average | B average | C average |  | A-B recovery rate | 106.9% |
| --- | --- | --- | --- | --- | --- |
| 510 | 545 | 525 |  | **A-C recovery rate** | 102.9% |

MS2 bacteriophage

Table 5. Plaque forming unit (PFU) counts recovered from (A) distilled water, (B) neutraliser, and (C) neutraliser with a copper coupon added, after inoculation with MS2 bacteriophage. Additionally, phage assays of the bacteriophage inoculum was performed to confirm initial virion concentration. All counts were performed from a pure (zero) dilution of the respective samples and a 10^-2^ dilution of inoculum. Averages of samples are displayed as per mL.

| Test | Repeat | Colony count | CFU / mL |
| --- | --- | --- | --- |
| Inoculated water (A) | 1 | 46 | 460 |
| Inoculated water (A) | 2 | 56 | 560 |
| Inoculated neutraliser (B) | 1 | 57 | 570 |
| Inoculated neutraliser (B) | 2 | 52 | 520 |
| Inoculated neutraliser with coupon (C) | 1 | 57 | 570 |
| Inoculated neutraliser with coupon (C) | 2 | 48 | 480 |
|  |  |  |  |
| Inoculum spread plates | 1 | 248 | 24800 |
| Inoculum spread plates | 2 | 237 | 23700 |
| Inoculum spread plates | 3 | 271 | 27100 |

| A average | B average | C average |  | A-B recovery rate | 106.9% |
| --- | --- | --- | --- | --- | --- |
| 510 | 545 | 525 |  | **A-C recovery rate** | 102.9% |

**References**

European Committee for Standardisation (2019) ‘EN 1276:2019 - Quantitative suspension test for the evaluation of bactericidal activity of chemical disinfectants and antiseptics used in food, industrial, domestic, and institutional areas.’
