# Supplementary figures and images for: Assessing antibacterial, antiviral, and antifungal efficacy of non-porous materials using small droplet transfer: the simulated splash method
Source: Appl Environ Microbiol. 2026 Apr 1;92(4):e02304-25. doi: 10.1128/aem.02304-25 (PMC13101517; doi:10.1128/aem.02304-25)

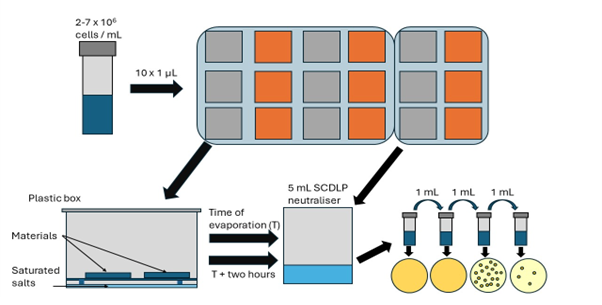

Supplement: Graphical abstract — Visual depiction of the study. [file aem.02304-25-s0003.tif]
